# Parosteal lipoma of the forearm: A case report and a literature review

**DOI:** 10.1097/MD.0000000000027876

**Published:** 2021-11-19

**Authors:** Asma’a Al-Mnayyis, Sarah Al Sharie, Mohammad Araydah, Muna Talafha, Fadi Haddad

**Affiliations:** a Department of Clinical Sciences, Faculty of Medicine, Yarmouk University, Irbid, Jordan; b Faculty of Medicine, Yarmouk University, Irbid, Jordan; c Princess Basma Teaching Hospital, Irbid, Jordan.

**Keywords:** Benign tumor, Case report, Forearm, Parosteal lipoma, Periosteal lipoma

## Abstract

**Rationale:**

Parosteal lipomas are rare neoplasms comprising mature adipocytes situated in a proximity to bone. Although these tumors follow a benign course, the reactive osseous changes that may occur with such lesions might raise the suspicion of malignancy.

**Patient Concerns:**

Here we present a case of a 33-year-old male patient complaining of pain and swelling in the right anterior forearm without history of trauma.

**Diagnosis:**

An magnetic resonance imaging of the region revealed a lobulated intramuscular fat intensity mass within the supinator muscle. Bony projection inseparable from the anterolateral radial diaphyseal cortex and periosteum was also seen. The radiological features suggested the diagnosis of parosteal lipoma.

**Intervention:**

After the radiological diagnosis of a parosteal lipoma, the patient was offered a total surgical excision of the mass.

**Outcomes:**

The mass was removed successfully. Histopathology showed mature benign adipose tissue bordered by thin fibrous septa confirming the diagnosis of parosteal lipoma. Follow-up magnetic resonance imaging after 6 months did not reveal any signs of complications or recurrence.

**Lessons:**

Distinction of the features of parosteal lipomas is needed to establish the accurate diagnosis, discriminate it from malignant lesions, predict potential neurovascular compromises, and follow up until a curative action is planned.

## 1 Introduction

Among tumors arising from soft tissues, lipomas are the most common benign form.^[1]^ Lipomas are classified according to their anatomical location into superficial lipomas (within the subcutaneous tissue) and deep lipomas (beneath the fascia).^[2]^ Deep lipomas can be further classified into intermuscular, intramuscular, or parosteal.^[3]^ Parosteal lipomas are benign tumors mostly solitary, arising from mature adipose tissue near the periosteum of bones and they account for <0.3% of all lipomatous lesions.^[4–6]^ Parosteal lipomas, as in other lipomas, are more frequent in the middle aged population (40–60 years’ old) and affect males and females equally.^[7,8]^ Most patients with parosteal lipomas are asymptomatic, although they might complain from a slowly growing mass or swelling. However, pain, sensory disturbances and weakness may be encountered if neurovascular structures are compromised.^[9–11]^ Although x-ray and computed tomography (CT) scans are considered useful in detecting and diagnosing parosteal lipomas, Magnetic resonance imaging (MRI) is the most preferable method in evaluating such tumors.^[12,13]^ Parosteal lipomas are becoming of significant importance as half of patients having this type of tumors possess accompanying bony lesions that may be suspected to be malignant.^[14]^ In this article we present a rare case of parosteal lipoma of the proximal forearm accompanied with a literature review of similarly reported cases in the English-based literature. This case has been reported in line with the CARE criteria.

## 2 Case presentation

**Figure 1:**
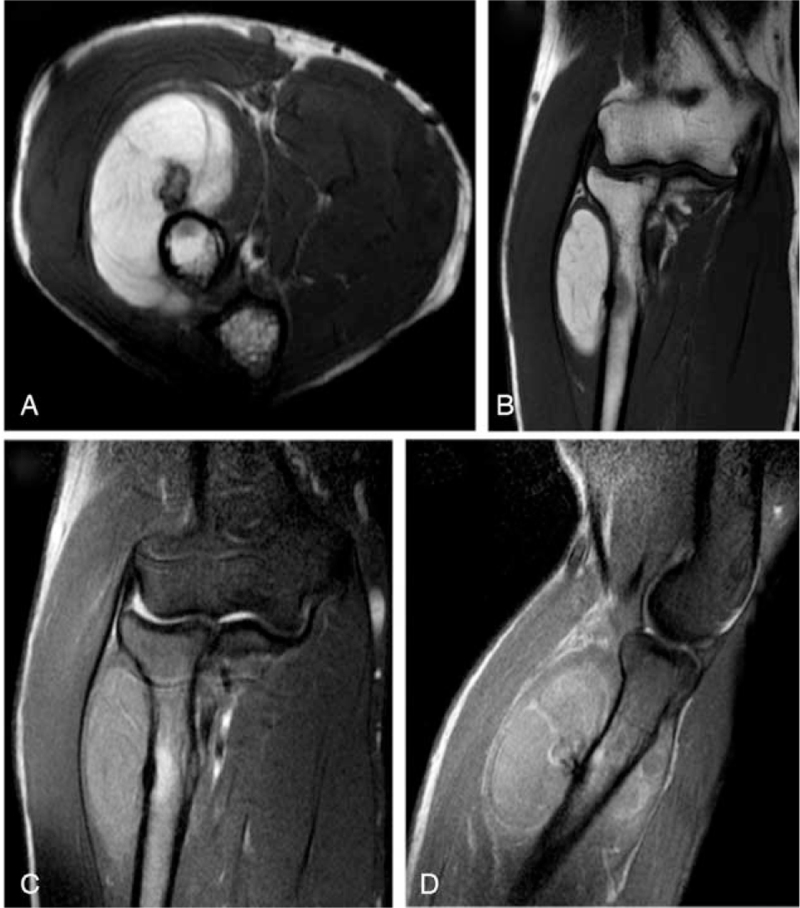
Parosteal lipoma of the proximal anterolateral forearm. Axial T1-weighted (A), coronal T1-weighted (B), coronal proton density (PD) fat saturated (C), and sagittal PD fat saturated (D) MRI of the right elbow and forearm demonstrate a lobulated parosteal lipoma with a bone protuberance projecting inside.

**Figure 2:**
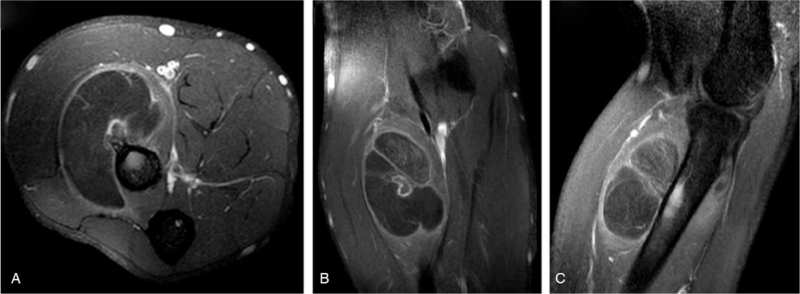
Parosteal lipoma of the proximal anterolateral forearm. Axial fat saturated T1-weighted (A), coronal fat saturated T1-weighted (B), and sagittal fat saturated T1-weighted (C) MRI of the right elbow and forearm after contrast administration showed enhancement of the septae and periosteum within the marginally enhanced parosteal lipoma.

**Figure 3:**
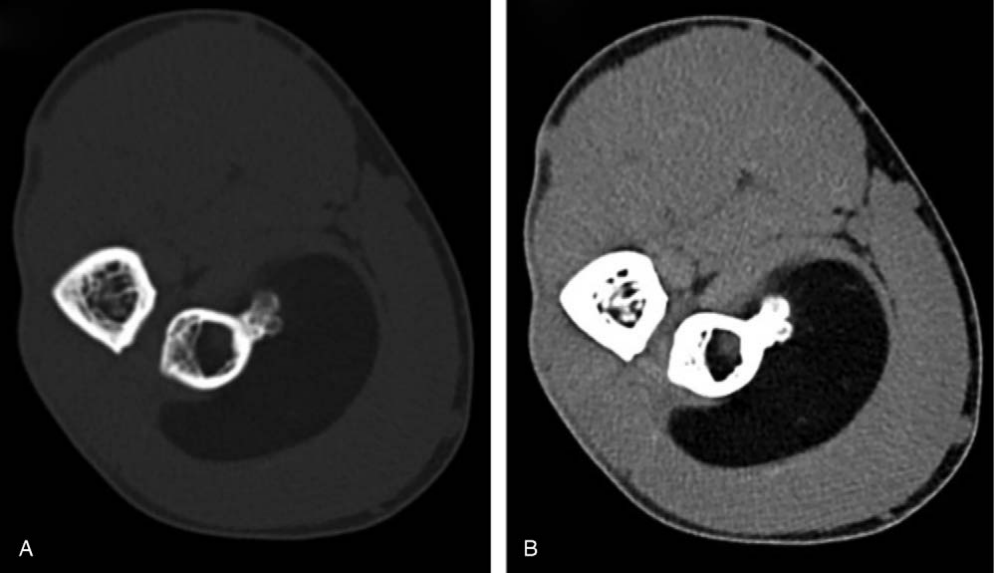
Parosteal lipoma of the proximal anterolateral forearm with internal radial bone protuberance. Axial bone window (A) and soft tissue window (B) computed tomography scan of the right forearm.

**Figure 4:**
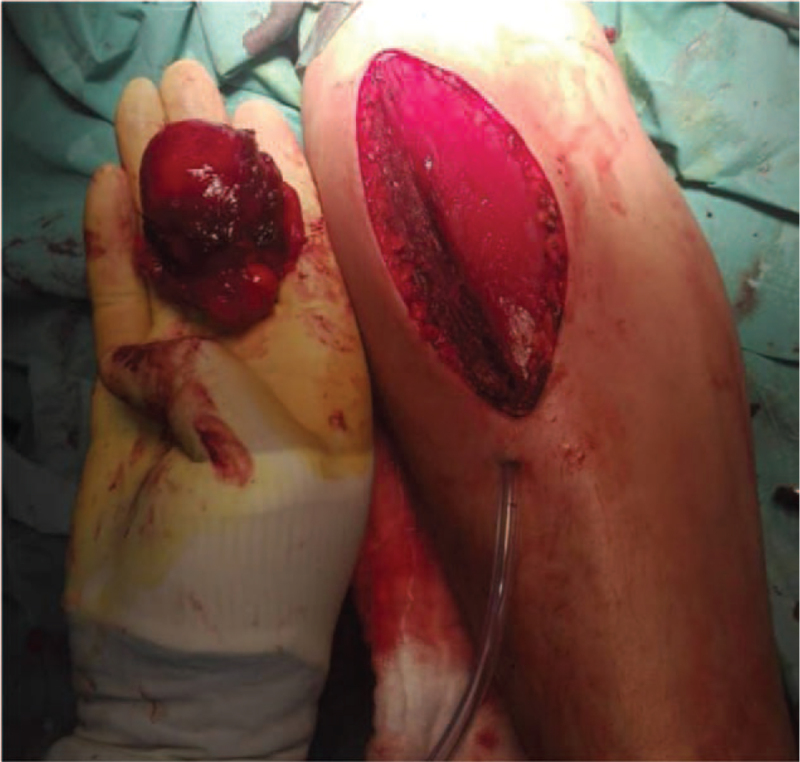
Intraoperative image of the excised tumor.

A 33-year-old male presented to our clinic, complaining of pain, and swelling in the right proximal anterior forearm (near the right elbow) that started after performing some sports. There was no history of trauma. Chronic or medical history, family history of chronic diseases, and drug history were all insignificant. On physical examination, a firm immobile swelling in the right anterior forearm was noticed. No skin discoloration, scars, or dilated veins were seen. Pain and tenderness at the site of the lesion were aggravated by wrist extension. Movement of the right elbow was within normal limits. No limitations in extension of the right wrist or fingers were detected. Radial pulses were palpated and bilaterally equal. An MRI was ordered to evaluate the detected mass and showed in (Fig. 1) a large intramuscular lobulated fat intensity within the supinator muscle and in a direct relation to the anterolateral proximal radial meta-diaphysis, containing bone protuberance inseparable from the anterolateral radial diaphyseal cortex and periosteum. The lesion contains thin septations inside and measures about 4 × 3 × 5.6 cm (in its maximum diameters). The radial cortex is slightly thickened with subcortical bone marrow edema noted. The mass is displacing the extensor muscles. There was no enhancement of the lesion itself but enhancing septations and periosteal layer of the protruding bone and the underlying bone marrow (Fig. 2). The posterior interosseous nerve (PIN) was not identified; mostly entrapped and compressed by the mass. Flexor and extensor muscles of the elbow appeared normal with no atrophy. Medial and lateral collateral ligaments appeared intact. There was no significant elbow joint effusion. A CT scan was done and confirmed the findings including the bony changes (Fig. 3). The patient underwent a successful surgical excision of the mass under general anesthesia and sterile technique. The surgery went through Henry‘s approach of the forearm, the proximal half of it was used as a plan A before extending it more distal if needed as plan B, Plan A was enough for this case. The right forearm was fully supinated on the arm board. The skin incision started from a point just lateral to biceps tendon till the midpoint of a line directed toward radial styloid process. Superficial dissection was started distally in the wound and was made between brachioradialis, and flexor carpi radialis then continued proximally between pronator teres and brachioradialis. Superficial radial nerve was isolated and the recurrent leash that arises from radial artery was ligated. Lateral retraction of the brachioradialis with superficial radial nerve was done, whereas Flexor Carpi Radialis and Pronator Teres with radial artery and its accompanying venae comitantes were medially retracted, revealing the supinator muscle and the associated mass. Supinator muscle was separated from its broad attachment and en bloc resection of the rounded encapsulated mass with its stalk was carried out. Grossly the mass measured around 4.3 × 3.1 × 5.4 cm, and it had a bony protuberance (Fig. 4). Histopathological examination showed mature benign adipose tissue bordered by thin fibrous septa confirming the diagnosis of parosteal lipoma. Postoperatively, the neurovascular examination was normal. A follow-up MRI was ordered 6 months later and showed no signs of complications. The management and prognosis of the case were fully explained for the patient in every step.

## 3 Literature review

### 3.1 Methods

A systematic thorough literature review following the Preferred Reporting Items for Systematic Reviews and Meta-Analysis guideline^[15]^ was conducted using 3 different databases (PubMed, Scopus, and Web of Science) via the key words and search terms “Parosteal Lipoma” OR “Periosteal lipoma.”

Two authors screened the obtained records from the selected databases independently by the title and abstract of the articles, then by full text. Records were included if they were case reports or case series reporting parosteal lipomas. Irrelevant studies or those written in languages other than English, or lacking full text, audits, letters to editor were all excluded. Different aspects of included cases were taken under consideration in the review such as, the publication year of the study, age, sex, symptoms of the lipoma, duration of symptoms, diagnostic methods used, location of the lipoma, measurements of the lipoma, presence of nerve compression, method of treatment, histopathological findings, and the follow-up period. Conflicts between authors in screening and data extraction were solved by the senior author of the study.

All included case reports and case series for data extraction were assessed for quality using the Joanna Briggs Institute critical appraisal tool for case reports and case series.^[16]^ Case reports were critically appraised by 8 questions concerning the demographics of the study, history description, clinical condition description, diagnostic tests or assessment methods, intervention of used procedure, post intervention clinical condition, and description of adverse events. Case series were critically appraised by 10 questions about the inclusion criteria and methods, reliability of condition measurement, validity of identification methods, demographics of the study, clarity of reported information, sufficiency of reported outcomes, and statistical analysis efficacy. In the quality assessment of both the case reports and case series, questions were answered by (yes), (no), or (unclear). Risk of bias for the study was determined based on the percentage of (yes) answered questions. A study was at high risk of bias if the percentage of (yes) answered questions was ≤49%, at moderate risk of bias if the percentage of (yes) answered questions was between 50% and 69%, and at low risk of bias if the percentage of (yes) answered questions was ≥70%.

## 4 Results

**Figure 5:**
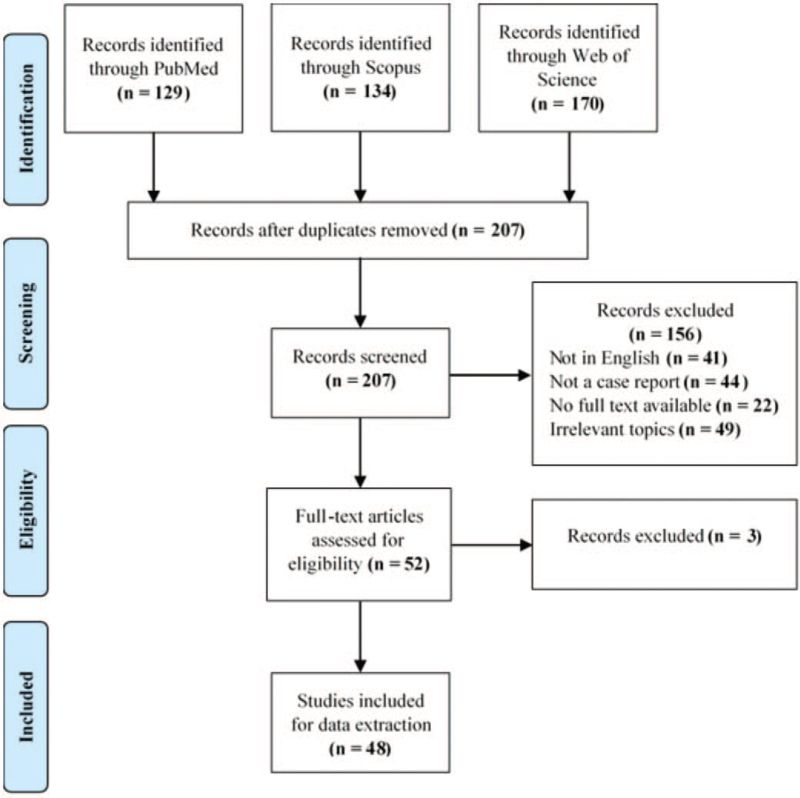
PRISMA flow diagram of the article selection process.

The search of parosteal lipoma cases in 3 different databases resulted in 433 records. A total of 226 duplicates were identified and removed. After title abstract screening and full text screening, 48 studies were finally included for data extraction (Fig. 5).

**Figure 6:**
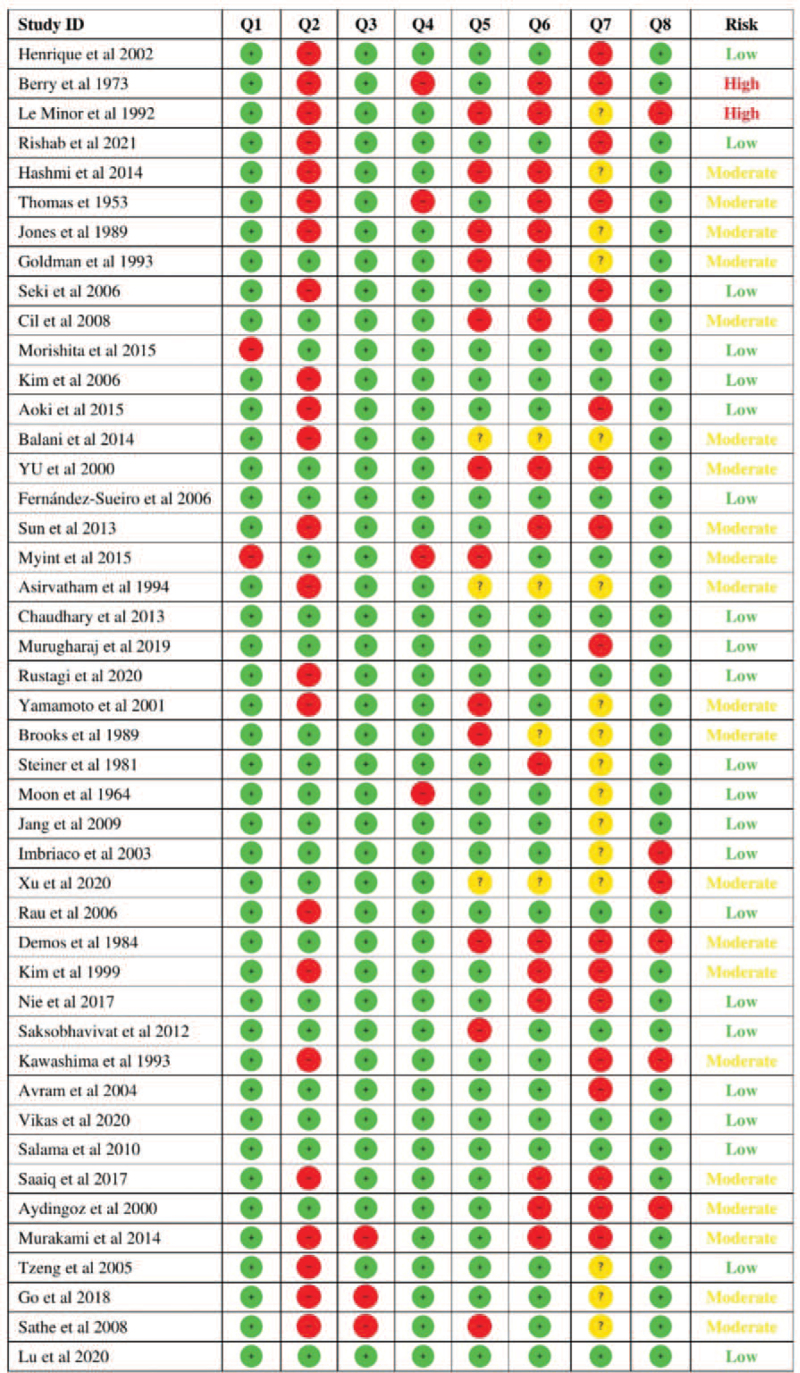
Risk of bias summary for included case reports.

**Figure 7:**
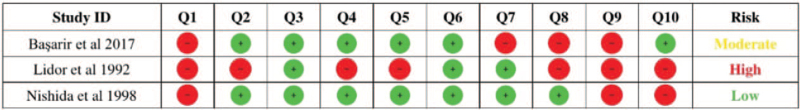
Risk of bias summary for included case series.

After screening obtained articles and assessing the methodological quality of the included studies using the Joanna Briggs Institute critical appraisal tool for case reports and case series by using the information provided by each article and referring to the authors of the studies in case of insufficient data, 3 studies were at high risk of bias (6.25%), 22 studies were at moderate risk of bias (45.83%), and 23 studies were at low risk of bias (47.91%). Figures. 6 and 7 demonstrate the risk of bias summary of included case reports and case series.

Different features of included case reports and case series were extracted and are summarized in (Table 1). The review included 64 patients reported in case studies and case series; 53.13% were females and 46.87% were males with a mean age of 49.25 (5–83 years’ old).

**Table 1:** Literature review of case reports data summary.

No.	Study ID	Year of publication	Age	Sex	Symptoms	Duration of symptoms, mo	Diagnostic methods	Location of the lipoma	Measurements of the lipoma, cm	Nerve compression	Follow-up period, mo
1	Henrique et al 2002	2002	57	F	Sensory loss, weakness (Rt hand)	—	x-Ray, ultrasound, MRI	Rt humerus	9 × 7 × 4	Radial nerve	1
2	Berry et al 1973	1973	30	M	Weakness (Rt hand)	18	Physical examination, EMG, and surgical exploration	Rt forearm	—	Radial nerve	—
3	Le Minor et al 1992	1992	67	M	Pain (Lt hand)	1/4	x-Ray, CT	Lt first metacarpal	1.9 In diameter	—	—
4	Rishab et al 2021	2021	47	M	Pain, swelling (Rt forearm)	12	x-Ray, MRI	Rt radius	4 × 4 × 6	Posterior interosseous nerve	3
5	Hashmi et al 2014	2014	45	F	Difficulty in walking and maintaining up right posture during sitting	18	x-Ray, MRI	Femur	8 × 6.5 × 14	—	—
6	Thomas et 1953	1953	58	M	Weakness, swelling (Rt forearm)	3/4	x-Ray	Rt radius	—	Posterior interosseous nerve	—
7	Jones et al 1989	1989	32	M	Painless mass (Rt thigh)	240	CT, MRI	Rt femur	18 × 13 × 8.5	—	—
8	Goldman et al 1993	1993	53	F	Swelling (Lt leg), 16 lb. weight loss	60, 12, and recent respectively	x-Ray, CT, MRI	Lt calf	11 × 7 × 5, 9 × 6 × 7	—	—
9	Seki et al 2006	2006	66	F	Painless mass (Rt leg)	—	x-Ray, MRI	Rt fibula	—	Common peroneal nerve	48
10	Cil et al 2008	2008	20	M	Mass (forehead)	36, 1 1/4	CT	Frontal bone	7 × 4.5	—	—
11	Morishita et al 2015	2015	5	M	Swelling (forehead)	48	CT, MRI	Frontal bone	—	—	24
12	Kim et al 2006	2006	57	F	Mass (posterior chest wall), progressive intercostal neuralgia	36	CT	Lt 6th rib	9 × 6 × 4	6th Intercostal nerve	2
13	Aoki et al 2015	2015	64	M	Rapidly growing mass (Rt thigh)	2	x-Ray, MRI	Rt femur	18 × 13 × 6	—	6
14	Balani et al 2014	2014	38	M	Painless swelling (Lt upper back)	36	x-Ray, MRI	Lt scapula	4.5 × 5.5 × 6.0	—	—
15	Yu et al 2000	2000	37	M	Asymptomatic	—	x-Ray, MRI	Lt femur	7.5 × 2.5 × 4.5	—	—
16	Fernández-Sueiro et al 2006	2006	52	M	Pain, swelling (wrists, hands, ankles and feet)	6	Bone scan, x-ray, CT, MRI	Both wrists	—	—	72
17	Sun et al 2013	2013	48	M	Slow-growing mass (chin), occasional numbness (Rt lower lip)	240	CT	Mandible	7 × 5 × 5	—	—
18	Myint et al 2015	2015	–	M	Slowly growing painless mass (thoracic spine)	36	Ultrasound	Thoracic spine (T4, T5, T6 spinous processes)	7.8 in diameter	—	—
19	Asirvatham et al 1994	1994	40	F	Gradually increasing swelling, discomfort (Rt thigh)	—	x-Ray, CT, MRI, bone scan	Rt femur	—	—	18
20	Başarir et al 2017^∗^	2017	46	M	Pain, swelling	60	MRI	Rt humerus	13 × 8 × 8	1 Case: the brachial plexus, 4 cases: the radial nerve, the rest had no nerve compression	16
			10	F	Swelling	12	MRI	Rt tibia	6 × 6 × 6		
			40	M	Swelling	36	CT, MRI	Lt forearm	7 × 5 × 5		
			50	F	Swelling	84	MRI	Lt radius	9 × 6 × 4		
			39	F	Pain, swelling, muscle weakness	12	MRI	Rt forearm	10 × 3 × 3		
			46	F	Swelling	18	MRI	Rt humerus	10 × 6 × 3		
			62	F	Pain, swelling	240	CT, MRI	Rt humerus	6 × 4 × 1		
			33	M	Pain, Swelling	60	MRI	Rt forearm	7 × 5 × 3		
			46	F	Pain, swelling	24	MRI	Lt humerus	10 × 8 × 4		
			57	F	Pain, swelling	96	MRI	Rt thigh	10 × 8 × 6		
			56	F	Pain, swelling	3	MRI	Lt humerus	5 × 5 × 7		
			60	M	Numbness (shoulder)	4	MRI	Rt humerus	10 × 7 × 5		
21	Chaudhary et al 2013	2013	65	F	Mass (Rt arm)	12	x-Ray, MRI	Rt humerus	12 × 5 × 8	—	—
22	Murugharaj et al 2019	2019	55	M	Progressively growing mass (Rt forearm)	360	x-Ray, MRI	Rt radius	8 × 8 × 4.5	—	24
23	Rustagi et al 2020	2019	32	F	Painless swelling (Rt forearm)	12	x-Ray, MRI	Rt radius	4.8 × 3 × 2	—	12
24	Yamamoto et al 2001	2001	68	F	Painless mass (Rt small finger)	36	x-Ray, CT, MRI	Rt small finger	12 × 8 × 8	—	28
25	Brooks et al 1989	1989	64	M	Gradually enlarging Rt index finger mass.	360	x-Ray, CT	Rt index finger	—	—	—
26	Steiner et al 1981	1981	50	M	Pain, Swelling (mandible)	3/4	x-Ray	Mandible	1.5 In diameter	—	—
27	Moon et al 1964	1964	62	F	Painless mass (Lt forearm)	24	x-Ray	Lt radius	6 × 3.5 × 3.5	Posterior interosseous nerve	60
28	Lidor et al 1992^∗^	1992	72	F	Complete extensor paralysis	24	x-Ray	Radius	—	2 cases: Posterior interosseous nerve, 2 cases: superficial radial nerve, 1 case: no nerve compression	—
			40	F	Complete extensor paralysis	4	x-Ray	Radius	—		—
			53	F	Painful growing tumor with paresthesia of ring finger	10	x-Ray	Radius	—		
			55	F	Slow-growing mass	3	x-Ray	Radius	—		
			40	F	Painful growing tumor with paresthesia of fingers	8	x-Ray, CT	Rt radius	4 × 3.5 × 4		
29	Jang et al 2009	2009	50	M	Rt pleuritic pain accompanied by acute respiratory symptoms, such as cough, sputum, and fever	2	x-Ray, CT	Rt 7th rib	8 × 6 × 2.5	—	4
30	Imbriaco et al 2003	2003	60	M	Progressive painful mass (Rt posterior upper chest)	4	CT	Rt 4th rib	5 × 3.5	—	26
31	Xu et al 2020	2020	59	M	Painless swelling (Rt upper back)	—	x-Ray, CT, MRI	Rt scapula	3.2 × 7.6 × 6.6	—	—
32	Rau et al 2006	2006	70	M	Mass (Lt thigh)	—	MRI	Lt femur	9.0 × 7.5 × 4.0	—	12
33	Demos et al 1984	1984	51	F	Mass (Lt arm), pain, numbness (Lt hand)	60	x-Ray, CT	Lt humerus	6 In diameter	—	—
34	Kim et al 1999	1999	46	F	Progressive painful mass (Lt thigh)	7	x-Ray, MRI	Lt femur	3 × 3	—	—
35	Nie et al 2017	2017	40	M	Slowly growing mass (Rt clavicle)	240	x-Ray, MRI	Rt clavicle	3 × 2 × 2	—	—
36	Saksobhavivat et al 2012	2012	35	F	Mass (Lt leg)	3	x-Ray, MRI	Lt fibula	3.5 × 3.0 × 3.0	—	4
37	Kawashima et al 1993	1993	44	F	Mass (Rt leg)	24	x-Ray, CT, MR	Rt tibia	4.5 × 3 × 2	—	4
38	Avram et al 2004	2004	69	M	Progressive difficulty with extending left long and ring fingers	4	—	Lt forearm	5 in length	Posterior interosseous nerve	8
39	Vikas et al 2020	2020	54	F	Progressive weakness of the right-hand extensors including thumb	5	x-Ray, MRI	Rt radius	—	Posterior interosseous nerve	7
40	Nishida et al 1998^∗^	1998	60	F	Inability to extend (Lt hand)	2	CT, MRI	Lt radius	6.5 × 4.5 × 2.0	Posterior interosseous nerve	40
			61	F	Painless Mass (Lt forearm)	—	x-Ray, CT, MRI	Lt radius	4.5 × 4.2 × 2.8	—	15
41	Salama et al 2010	2010	83	F	Acute and progressive weakness (Rt-hand extensors), painless swelling (Rt forearm)	1 1/2	x-Ray, MRI	Rt radius	—	Posterior interosseous nerve	6
42	Saaiq et al 2017	2017	53	M	Weakness of extension of the fingers and the thumb (Lt hand)	7	MRI, electromyography, nerve conduction studies	Lt radius	6 × 5 × 4	Posterior interosseous nerve	4
43	Aydingoz et al 2000	2000	39	F	Mass (Lt thigh)	4	x-Ray, CT, MRI	Lt femur	—	—	—
44	Murakami et al 2014	2014	50	F	Mass (Lt parieto-occipital portion)	240	CT, MRI	Skull	5 × 8	—	—
45	Tzeng et al 2005	2005	56	F	Pain (Lt forearm), numbness, pain (Lt thumb)	2	x-Ray, CT, ultrasound	Lt radius	3 × 2 × 2.3	Superficial radial nerve	15
46	Go et al 2018	2018	33	M	Mass (chest wall)	24	CT, MRI	Rt 4th rib	3 in diameter	—	—
47	Sathe et al 2008	2008	20	M	Slow growing mass (Rt side of chest)	168	CT	Rt 4th rib	5 × 3 × 2.5	—	—
48	Lu et al 2020	2020	70	M	Mass (Lt arm)	288	x-Ray, MRI	Lt humerus	2.7 × 1.9 × 0.9	—	60
49	Present case	2021	33	M	Pain, swelling (Rt forearm)	—	CT, MRI	Rt humerus	4.3 × 3.1 × 5.4	Posterior interosseous nerve	6

∗ Case series. CT = computed tomography, EMG = Electromyography, F = female, M = male, MRI = magnetic resonance imaging, Rt = right, Lt = left.

The forearm was noticed to be the most common location of parosteal lipomas in the reviewed studies as it appeared in 22 patients (34.38%), followed by the arm in 10 patients (15.63%). The presence of a parosteal lipoma in the clavicle, scapula, forehead, and the spine was extremely rare.

Of the 32 patients with parosteal lipomas located in the arm or forearm, 9 patients had a PIN compression, nine had a radial nerve compression and 1 patient had a brachial plexus compression. Forty-three patients of the overall number did not report any nerve compression.

Regarding the treatment of the parosteal lipoma patients of the review, 60 of them had a complete surgical resection of the tumor (93.75%), 2 patients were conservatively treated (3.125%) and 2 patients refused the surgery (3.125%).

Histopathological examination was performed for fifty59 patients of those who underwent surgeries and confirmed the diagnosis of parosteal lipoma by observing mature fat cells without cellular atypia or lipoblasts.

Follow-up periods of patients were not reported in twenty-four cases. The average follow-up period of the remaining studies was 19.44 months. None of the included studies mentioned any signs of tumor recurrence.

## 5 Discussion

Lipomas are the most common benign mesenchymal tumors and are composed of adipose cells typically arising in soft tissues, affecting 1 of each 1000 persons and have a higher incidence to occur in males than in females. They can originate in any place where there are adipocytes.^[17,18]^

Lipomas of the bone are considered less frequent than other types of lipomas as they comprise <0.1% of all bone tumors.^[19]^ When lipomas are closely related to bones, they are called osseous lipomas which are divided according to their location of placement into intraosseous (within the bones) and Parosteal (on the surface of the bone).^[20]^

The first publication of a parosteal lipoma case in the English based medical literature took place in 1866 by Smith et al in which it was described as a “Specimen of firm bilobed fatty tumor.”^[21]^ Until 1888, the term periosteal lipoma was sometimes used in the medical literature but, in his publication, entitled “A Parosteal lipoma, or congenital fatty tumor connected with the femur” in 1888, Power et al asserted the origin of those tumors and introduced the term “Parosteal lipoma” that is still being used until these days. The term periosteal was replaced by parosteal to emphasize the idea that this kind of tumors does not necessary originate from the bone itself but can be adjacent to it.^[22]^

Parosteal lipomas are defined as benign rare tumors composed of mature adipose tissue closely related to the periosteum, usually asymptomatic, accounting for 0.3% of all lipomas and mostly affecting middle aged patients (older than 40 years) and are equally frequent in both females and males.^[7,8,23]^

Parosteal lipomas usually develop in the diaphysis of long bones. The most common sites of parosteal lipomas in order are the femur, radius, tibia, and then the humerus.^[24]^ The presence of such tumors in small bones such as the carpals is extremely rare.^[25]^

A parosteal lipoma usually presents as a palpable, slowly growing, large, immobile mass.^[26]^ Depending on the size, location of the tumor, and the presence of adjacent neuromuscular or neurovascular structures, nerve compressions may develop thus causing deficits in sensory or motor functions and leading to muscle atrophy. Many cases, as in our case, reported the compression of the PIN due to parosteal lipomas of the forearm.^[27,28]^

On x-ray, a parosteal lipoma appears as a radiolucent fat containing mass that is well circumscribed with an intimate relation to the bone's periosteum. This typical characteristic finding may be difficult to appear if the mass is located in an area overlayed with osseous structures as in the pelvis.^[29]^ Reactive changes of the bone surrounding parosteal lipomas may also be seen. These reactions include cortical thickening, calcification, or sclerosis. Smooth scalloping of the cortex, or osseous bowing or projection, can be also visualized.^[30]^

On non-contrast CT scans, these lesions appear as well-demarcated masses of low density (−120 to −60 Hounsfield unites).^[31]^ Using MRI, parosteal lipomas will be shown as lesions with signal intensity like that of subcutaneous fat.^[32]^ On some occasions, alongside bone projections, hyaline cartilages of intermediate (on T1 sequence MRI), or high (on T2 sequence MRI) signal intensities may be visualized. Fibrous tissues, may also be seen, they can be differentiated from cartilages by their low signal intensity appearance on T2 sequence MRIs even after contrast enhancement.^[33]^ Linear streaks of high signal intensity within muscles on all MRI pulse sequences representing fat can be an indicator to muscle atrophy due to nerve compression.^[34]^

In planning for surgical excision, the relationship between osseous excrescence and lipomatous mass with adjacent muscles and underlaying bony cortex is better visualized on MRI due to its callability of multiplanar imaging.^[29]^ CT and MRI are also useful in differentiating osseous projections in parosteal lipomas from osteochondromas because medullary continuities with adjacent bone are absent in parosteal lipomas.^[33]^

In this type of lipomas, the optimal method of management is complete surgical excision of the tumor, especially in cases wherein certain nerve compression by the mass is present, to reduce the chances of permanent nerve damage or muscle atrophy.^[35]^ To avoid recurrence of the lipoma, wide and complete combined margin excision are suggested.^[36]^ Histopathological findings of mature adipocytes without cellular atypia can confirm the diagnosis of parosteal lipomas after the surgical excision.^[37]^

## 6 Conclusions

The clinical approach to parosteal lipomas requires collaborative efforts from physicians in multiple disciplines to diagnose accurately and treat promptly, as rapid restoration of patients’ quality of life remains the top priority in management. Distinction of the features of such lesions using MRI which is the criterion standard modality is vital, to establish diagnosis, discriminate it from malignant lesions, predict potential neurovascular compromises, and follow-up until a curative action is planned.

## Author contributions

We would like to thank our patient for consenting to the publication of this article.

**Conceptualization:** Asma’a Al-Mnayyis, Mohammad Araydah.

**Data curation:** Sarah Al Sharie, Mohammad Araydah, Muna Talafha, Fadi Haddad.

**Investigation:** Asma’a Al-Mnayyis.

**Methodology:** Sarah Al Sharie, Muna Talafha.

**Resources:** Sarah Al Sharie.

**Supervision:** Asma’a Al-Mnayyis.

**Validation:** Asma’a Al-Mnayyis.

**Visualization:** Asma’a Al-Mnayyis, Mohammad Araydah.

**Writing – original draft:** Asma’a Al-Mnayyis, Sarah Al Sharie, Mohammad Araydah, Muna Talafha, Fadi Haddad.

**Writing – review & editing:** Asma’a Al-Mnayyis, Sarah Al Sharie.
